# Tumour-derived exosomes as antigen delivery carriers in dendritic cell-based immunotherapy for malignant mesothelioma

**DOI:** 10.3402/jev.v2i0.22492

**Published:** 2013-10-24

**Authors:** Niken M. Mahaweni, Margaretha E.H. Kaijen-Lambers, Jacqueline Dekkers, Joachim G.J.V. Aerts, Joost P.J.J. Hegmans

**Affiliations:** Department of Pulmonary Medicine, Erasmus MC Cancer Institute, Rotterdam, The Netherlands

**Keywords:** mesothelioma, dendritic cell(s), immunotherapy, tumour antigens, exosomes

## Abstract

**Background:**

In 2001, it was postulated that tumour-derived exosomes could be a potent source of tumour-associated antigens (TAA). Since then, much knowledge is gained on their role in tumorigenesis but only very recently tumour-derived exosomes were used in dendritic cell (DC)-based immunotherapy. For this, DCs were cultured *ex-vivo* and loaded with exosomes derived from immunogenic tumours such as melanoma or glioma and re-administrated to induce anti-tumour responses in primary and metastatic tumour mouse models. In contrast, malignant mesothelioma (MM) is a non-immunogenic tumour and because only a few mesothelioma-specific TAA are known to date, we investigated whether mesothelioma-derived exosomes could be used as antigen source in DC-based immunotherapy.

**Methods:**

Mouse MM AB1 cells were used to generate tumour lysate and tumour-derived exosomes. Tumour lysate was generated by 5 cycles of freeze–thawing followed by sonication of AB1 cells. Tumour exosomes were collected from the AB1 cell culture supernatant and followed a stepwise ultracentrifugation. Protein quantification and electron microscopy were performed to determine the protein amount and to characterise their morphology. To test whether MM derived exosomes are immunogenic and able to stimulate an anti-tumoral response, BALB/c mice were injected with a lethal dose of AB1 tumour cells at day 0, followed by intraperitoneal injection of a single dose of DCs loaded with tumour exosomes, DCs loaded with tumour lysate, or phosphate buffered saline (PBS), at day 7.

**Results:**

Mice which received tumour exosome-loaded DC immunotherapy had an increased median and overall survival compared to mice which received tumour lysate-loaded DC or PBS.

**Conclusion:**

In this study, we showed that DC immunotherapy loaded with tumour exosomes derived from non-immunogenic tumours improved survival of tumour bearing mice.

Exosomes are 30–100 nm extravesicular membrane particles that are released by virtually all cells in the body as well as tumour cells (1, 2). Previous studies have described that exosomes are involved in many biological functions, such as intercellular communication, antigen presentation, protein secretion, RNA shuttling, depending on the cell of origin (3, 4). Exosomes secreted by tumour cells contain mRNA and proteins, among them chaperon proteins such as tetraspanins (e.g. CD9, CD63, CD81), heat-shock protein (Hsp) 70, major histocompatibility complex (MHC) class I molecules (5, 6), as well as tumour associated antigens (TAA), for instance Mart1, melan-A, gp-100 proteins, mesothelin, carcinoembryonic antigen and Her2-neu (7, 8).

Due to these potential immunogenic properties, studies have been performed to take advantage of tumour exosomes as an antigen source for dendritic cell (DC) immunotherapy in melanoma, renal cell carcinoma, and glioma (9–11). The outcome of these studies was encouraging. DCs loaded with tumour exosomes were able to cross-present the antigens to cytotoxic T cell (12), induce differentiation and expansion of tumour-specific cytotoxic T cells (11) and activate antitumor responses in these immunogenic tumours (6).

Malignant mesothelioma (MM) is traditionally considered as a non-immunogenic tumour, meaning that they have lost the ability to trigger a strong immune response by the host. Because not all known MM associated antigens expressed in all MM cases, our previous studies both in mice and patients (13, 14) used necrotic tumour lysate as a source of antigens to load DCs for DC-based immunotherapy to treat MM patients. To optimise this therapy, we continuously investigate the most efficient source of tumour antigen to load DCs. We found that MM released an abundant amount of exosomes (15). Our proteomic analysis to characterise the protein content of exosomes released by human mesothelioma cell lines, revealed that these exosomes are enriched with molecules that may be involved in antigen presentation, for example; MHC class I molecules and Hsps (16) although no mesothelioma-associated antigens were identified. However, another study done by Clayton et al. detected the presence of Her2-neu and mesothelin using Western Blotting techniques (17). These findings altogether indicate that the level of tumour antigens might be relatively low in human mesothelioma cell lines-derived exosomes.

Therefore, in this study, we investigated the feasibility of using mesothelioma cell line-derived exosomes to load DCs in a mouse mesothelioma immunotherapy model.

## Methods

### Cell line culturing and media

The AB1 mouse mesothelioma cell line was a gift from Prof. Bruce W. Robinson, Queen Elizabeth II Medical Centre, Australia. This highly immunosuppressive and non-immunogenic cell line was derived from BALB/c mice that developed mesothelioma after exposure to asbestos fibres. The cells were cultured in exosome-free medium (EFM) culture media consisting of RPMI 1640 supplemented with 25 mM HEPES, GlutaMax, 50 µg/ml gentamycin (all obtained from Invitrogen, Breda, the Netherlands) and 5% exosome-free foetal bovine serum (FBS) (HyClone, Thermo Scientific, United Kingdom). Exosome-free FBS was prepared by first heating the serum to 56°C for 30 minutes in a water bath to destroy heat-labile complement proteins. FBS was then centrifuged at 400×g for 10 minutes and at 2,000×g for 20 minutes (Hereaus Instruments). Finally, the FBS was centrifuged at 100,000×g (Ultracentrifuge Hereaus 5 M, SW 28 rotor) using ultra clear centrifuge tubes (Beckman Coulter, CA, USA) at 4°C for at least 16 hours to remove all vesicles and exosomes. The supernatant containing exosome-free FBS was stored at −20°C until use.

### Tumour exosome isolation

In order to obtain tumour exosomes, AB1 tumour cells were cultured in EFM to avoid the presence of vesicles from the supplements. Tumour exosomes were collected from the supernatant of AB1 cell culture equals to 100×10^6^ cells according to previously described ultracentrifugation methods (18). The supernatant from the cultured AB1 tumour cell line was centrifuged at increasing speeds to eliminate large cell debris and aggregates. Supernatant was sequentially centrifuged at 400×g for 10 minutes and 2,000×g for 20 minutes (Hereaus Instruments). Next, supernatant was centrifuged at 10,000×g at 4°C for 30 minutes to pellet cells and debris (High speed centrifuge Hereaus Instruments). Finally, the supernatant was ultra-centrifuged at 100,000×g for 1 hour to pellet the exosomes (Ultracentrifuge Hereaus 5 M, SW 28 or SW 32 rotor) using ultra clear centrifuge tubes (Beckman Coulter). The pellet was resuspended in phosphate buffered saline (PBS), frozen in liquid nitrogen and stored at −80°C.

### Tumour lysate production

AB1 cells from the cell culture were collected and resuspended at a concentration of 100×10^6^ cells/ml in PBS. AB1 tumour lysate was generated by 5 cycles of freezing viable cells in liquid nitrogen and thawing at room temperature followed by sonication (Branson Sonifier 250) for 10 seconds on ice followed by 50 seconds rest, repeated for 3 times, to produce a homogeneous lysate.

### Protein quantification

The CBQCA-protein quantification kit (Invitrogen) was used to determine the amount of protein contained in tumour exosomes and necrotic tumour lysate. The CBQCA protein quantification kit uses the ATTO-TAG CBQCA reagent (3-(4-carboxybenzoyl)quinoline-2-carboxaldehyde) for the quantitation of proteins in solution. The protein samples were diluted into a sodium borate buffer (47.5 mg/ml Na_2_B_4_O_7_ 10 H_2_O, pH 9.3). Potassium cyanide (KCN) and CBQCA were added to the solution to start the reaction with the primary amines to form highly fluorescent derivatives. After an incubation period of 2 hours, the samples were analysed in a fluorescence microplate reader (BMG labtech, Ortenberg, Germany) using excitation/emission wavelength of 465/550 nm, respectively. Bovine serum albumin (BSA) was used as a protein standard according to manufacturer's suggestion. The optical density (OD) of the dilution series was used to calculate a standard curve. The protein concentration was determined by using the standard curve and the OD value of the samples.

### Electron microscopy

Electron microscopy was performed at Dept. of Clinical Genetics of the Erasmus Medical Center, Rotterdam, The Netherlands, for samples of the necrotic tumour cell lysate and exosomes. Briefly, necrotic tumour cell lysate and exosome fractions were thawed and incubated on formar-coated grids for 15 minutes. After 3 washes with milli-Q water (Millipore Corporation, Etten-Leur, The Netherlands) for 2 minutes each, samples were negatively stained with uranyl acetate and examined with a Philips CM 100 electron microscope (EM) at 80 kV (Philips Industries, Eindhoven, The Netherlands).

### Generation of DCs and loading of DCs with tumour exosome or tumour lysate

DCs were generated using modification of a previously described procedure (19). Instead of flushing the mouse bone marrow, the whole femur and tibia were crushed using a pestle and mortar with the addition of approximately 3 mL of RPMI 1640 medium (Invitrogen). Cells were passed through a 100-µm cell strainer. On day 0, femurs and tibias were crushed and bone marrow cells were isolated and put in culture. DC-tissue culture medium was RPMI 1640 medium (Invitrogen) containing Glutamax-I (Invitrogen) supplemented with 5% exosome-free FBS, 50 µg/ml gentamycin, 50 µM β-mercaptoethanol (Sigma-Aldrich, MO, USA) and 20 ng/ml recombinant murine granulocyte macrophage-colony-stimulating factor (GM-CSF) (gift from Prof. Bart Lambrecht). We used 6-well plates to culture DCs, with a total number of 600,000 cells/well at seeding. On day 3, new tissue culture medium was added to the plates and cells were cultured for 8 days at 37°C (Innova Co-170, New Brunswick Scientific) in a humidified atmosphere at 5% CO_2_. After 8 days in culture, DCs were loaded with exosomes or tumour lysate and after 8 hours, 100 µg/ml Lipopolysaccharide (LPS) [E. coli Serotype R515 (Re): Enzo Life Science] was added to the DC culture to allow complete maturation while incubated overnight at 37°C. Exosome-loaded DCs or tumour lysate-loaded DCs were washed in PBS at 400×g for 7 minutes with a Varifuge 3. OR centrifuge (Heraeus Instruments) at 4°C and resuspended in 3 ml PBS. Next, these DCs were separated and purified from free tumour material using 3 ml lympholyte-Mammal (Cedarlane, Ontario, Canada) density gradient centrifugation. After centrifugation at 1,200×g (Heraeus Instruments) for 20 minutes at room temperature, DCs were collected from the interphase using a pipette. DCs were washed in PBS at 400×g for 7 minutes at 4°C and resuspended in a concentration of 1.6×10^6^ cells/500 µl PBS for *in vivo* DC immunotherapy.

### FACS analysis: maturation of stimulated DCs

DCs were analysed by the following markers: CD11c (PE Texas Red) and MHCII (AF700) CD40 (APC), CD80 (PerCP-Cy5.5), and CD86 (PE Cy7). DAPI was added to exclude dead cells and relevant isotype antibodies were used as control. Antibodies were diluted in a ratio of: 1:200 CD40, 1:800 CD80, 1:1,600 CD86, 1:400 MHCII, 1:100 CD11c and 1:300 2.4.G2 (to prevent non-specific binding). Live/dead marker DAPI (Invitrogen) was added in a 1:5,000 dilution. After 30 minutes of incubation at 4°C, cells were washed twice in binding buffer and the cells were subsequently measured on the FACS LSR II.

### In vivo experiment

Eighteen female BALB/c mice 6–8 weeks old (Harlan, Zeist, The Netherlands) were housed at the animal care facility of the Erasmus MC, Rotterdam. The local Ethical Committee for Animal Welfare approved the experiment. At day 0, all BALB/c mice were injected intraperitoneally with a lethal dose of 0.5×10^6^ AB1 tumour cells. On day 7, 6 mice received an injection with 500 µl PBS as control, 6 mice received DCs loaded with tumour lysate, and 6 mice received DCs loaded with tumour exosomes. Each vaccine contained 1.6×10^6^ DCs diluted in 500 µl PBS. Tumour growth, physical well-being, body temperature and survival were monitored up to 52 days after tumour injection. Mice were killed if profoundly ill according to UKCCCR regulations, and were scored as a death in survival analysis. At day 52, surviving mice were sacrificed.

### Immunohistology of tumour material

Tumour material was collected from mesothelioma bearing mice that were euthanised at day 15 (untreated mice) and day 52 (DC-treated mice). Tissue sections (7 µm) were cut on a HM-560 cryostat (Microm, Heidelberg, Germany) and washed in acetone for 10 minutes before adding Normal Goat Serum (1:10 diluted) in blocking buffer 1%. Immunostaining was performed using CD3 (clone 17A2, eBioscience), CD4 (clone 53–6.7, Serotec), CD 8 (clone RM4–5, Bioconnect) and CD11c (clone N418, BD Bioscience) antibodies, with a 1-hour incubation period. Binding of antibodies was detected using immune-alkaline phosphatase (AP) on the secondary antibodies Goat-anti-Rat (1:50) and Goat-anti-Hamster (1:50). Naphtol-AS-MX-phosphate (0.30 mg·mL^−1^, Sigma-Aldrich) and new fuchsine (160 mg·mL^−1^ in Sodium Nitrite) were used as substrate. Finally, the tissue sections were stained with Mayer's hematoxylin for nuclear staining.

## Results and discussion

### Mesothelioma-derived exosomes are immunogenic and prolong survival of tumour bearing mice

**Fig. 1:**
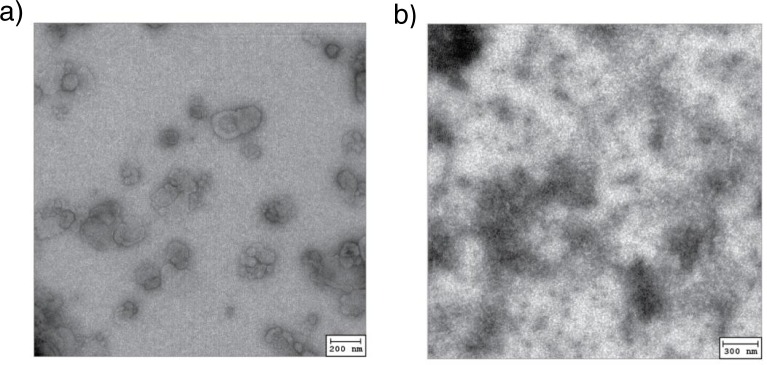
Morphology of AB1 tumour exosome (left) in comparison to necrotic AB1 tumour cell lysate (b) visualised by electron microscopy.

We isolated tumour exosomes from the culture supernatant of the murine mesothelioma AB1 cell line by ultracentrifugation. The morphology of the pellet obtained after ultracentrifugation was checked by electron microscopy. Freeze–thawed cell lysate was collected for comparison. We observed that the morphology of the pellet fits the description of exosomes as a rather homogeneous population of cup shaped vesicles ranging between 30 and 50 nm in diameter (1, 2), while tumour lysate appears as heterogeneous fragments >150 nm in size (Fig. 1).

**Fig. 2:**
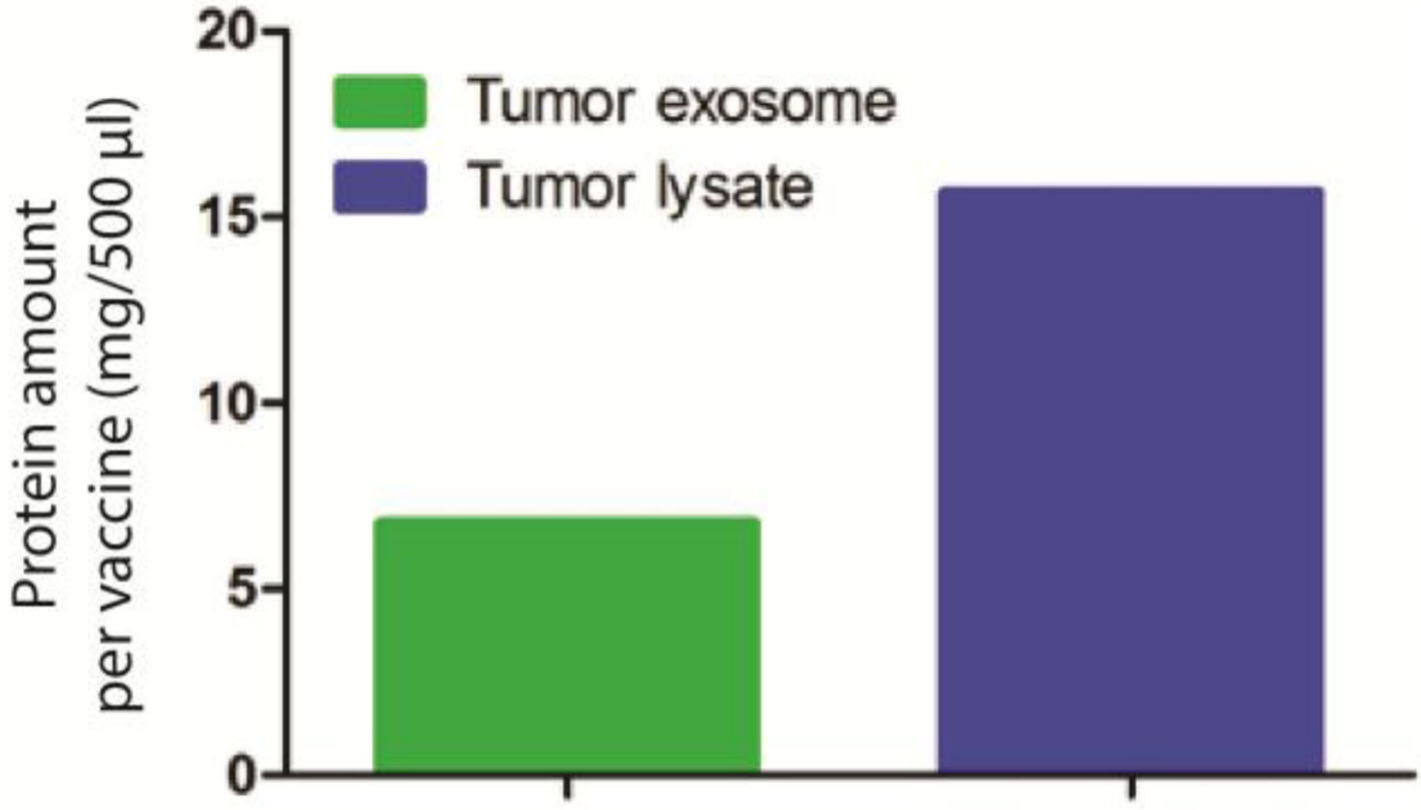
Protein amount analysis for tumour exosome (6.78 mg) used in DC immunotherapy compared to equivalent amount of necrotic tumour lysate (15.67 mg).

To investigate whether these exosomes are capable of inducing antitumor responses, we set up an *in vivo* experiment using BALB/c mice syngeneic to AB1 cell line. Seven days prior to DC immunotherapy, we injected a lethal dose of 0.5×10^6^ million AB1 cells to 12 mice. For the DC immunotherapy, we loaded AB1 tumour exosomes equivalent to 60×10^6^ AB1 tumour lysate to 10×10^6^ DCs *in vitro*. The protein amount of exosomes and tumour lysate were measured (Fig. 2) using CBQCA protein quantification kit, based on the ATTO-TAG CBQCA reagent (3-(4-carboxybenzoyl) quinoline-2-carboxaldehyde) that quantifies amines in solution, including accessible amines in proteins (20).

After an overnight stimulation with tumour exosomes or tumour lysate and LPS to ensure DC maturation (Fig. S1), we harvested DCs and injected 1.6×10^6^ tumour exosomes-loaded DCs to 6 tumour bearing BALB/c mice or 1.6×10^6^ tumour lysate-loaded DCs to 6 tumour bearing BALB/c mice. As a control, we injected 6 tumour bearing BALB/c mice with PBS.

**Fig. 3:**
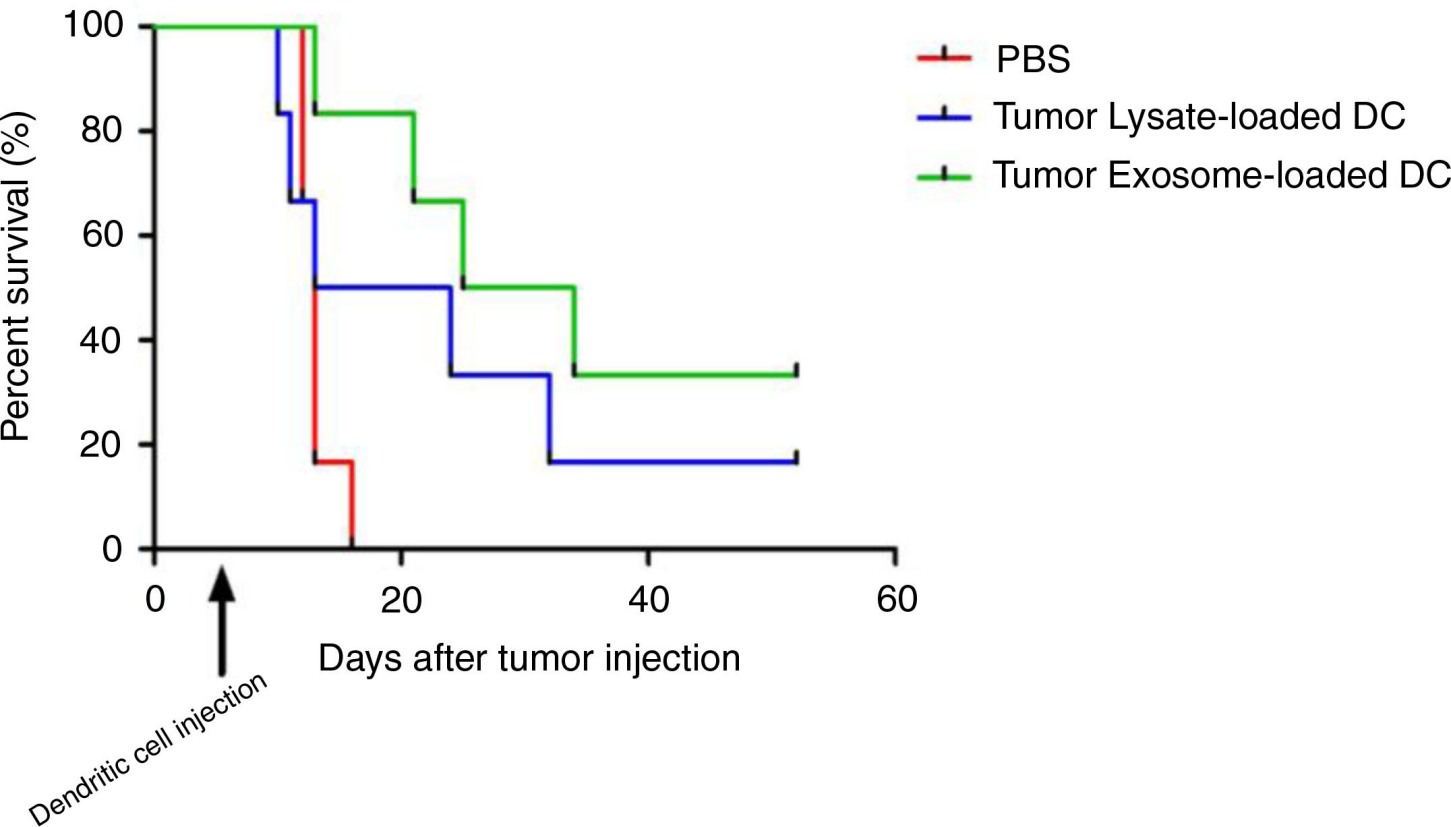
Kaplan Meier survival curve of mesothelioma bearing mice treated with tumour exosome loaded DC or tumour lysate loaded DC. Dendritic cell injection was administered 7 days after tumour injection.

In the survival curve, within 15 days after tumour injection, all mice from the PBS control group died In contrast, DC treated group had an increase in survival; 16.7% of mice from necrotic tumour lysate-loaded DC group and 33.3% of mice from tumour exosome-loaded DC group survived (Fig. 3) until the end of the experiment (52 days). The median survival for PBS group=13 day, tumour lysate-loaded DC group=18.5 day and exosome-loaded DC=29.5. Logrank (Mantel-Cox) test p-value=0.0992 (not significant). Logrank test for trend p=0.00342 (significant). Interestingly, from this survival curve, mice receiving tumour exosome-loaded DC displayed a better survival compared to mice receiving necrotic tumour lysate-loaded DC, which by definition contain whole tumour antigens.

**Fig. 4:**
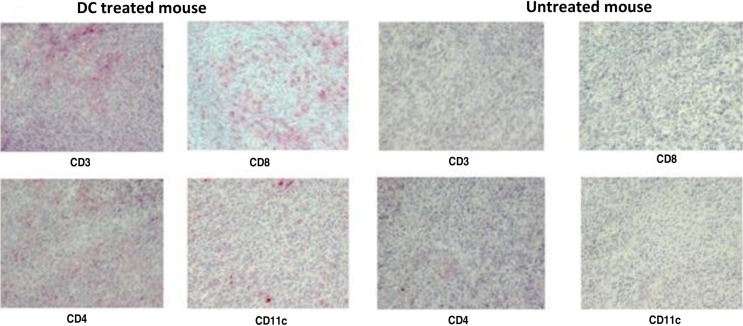
Immunohistology of tumour material taken from a mouse treated with DCs (representative for exosome and tumour lysate-DC group) (day 52) and a non-treated tumour bearing mouse (day 15). CD3, CD4 and CD8 were used to stain different subsets of T cells (positive cells=red). CD11c was used to stain DCs.

The surviving tumour exosome-loaded DC treated mice did develop tumours, but we noticed a remarkable difference between the tumours developed in these mice and tumours developed in control group. Immunohistochemistry results on the tumour isolated from the 2 groups demonstrated distinct cellular composition inside the tumour nest. We observed the presence of abundant CD4^+^ T cells, CD8^+^ T cells and DCs infiltration within the tumour of the DC vaccine treated group (Fig. 4). In contrast, this phenomenon was indiscernible within the tumours that developed in the control group.

Altogether, these findings suggest that mesothelioma exosome-loaded DC vaccine was capable of stimulating immune responses in mesothelioma bearing mice resulting in an increased survival compared to untreated group and the tumour lysate-loaded DC group.

Although in this experiment we did not include a group of mesothelioma bearing mice which received unloaded DC to show that this effect was indeed mesothelioma exosome dependent, our previous study (13) showed that unloaded DC do improve survival compared to no treatment but by far not as efficient as tumour lysate loaded DC.

## Conclusion

Exosomes derived from non-immunogenic and highly suppressive mesothelioma tumours are enriched with tumour antigens and are capable in inducing anti-tumour responses when presented by DCs to the T cells *in vivo*. Although mesothelioma-derived exosomes contained less proteins than necrotic tumour lysate (Fig. 2), when loaded to DCs, it improved the efficacy of DC immunotherapy.

To confirm the immunogenicity of mesothelioma-derived exosomes, future studies are required to further define the composition of these exosomes to optimise and standardise the technique. Here, we show that tumour exosomes from the non-immunogenic mesothelioma tumour improve the efficacy of DC-based immunotherapy for MM.

## References

[1] Kesimer M, Scull M, Brighton B, DeMaria G, Burns K, O'Neal W (2009). Characterization of exosome-like vesicles released from human tracheobronchial ciliated epithelium: a possible role in innate defense. FASEB J.

[2] Yang C, Robbins PD (2011). The roles of tumor-derived exosomes in cancer pathogenesis. Clin Dev Immunol.

[3] Van Niel G, Porto-Carreiro I, Simoes S, Raposo G (2006). Exosomes: a common pathway for a specialized function. J Biochem.

[4] Schorey J, Bhatnagar S (2008). Exosome function: from tumor immunology to pathogen biology. Traffic.

[5] Duijvesz D, Luider T, Bangma CH, Jenster G (2011). Exosomes as biomarker treasure chests for prostate cancer. Eur Urol.

[6] Wolfers J, Lozier A, Raposo G, Regnault A, Théry C, Masurier C (2001). Tumor-derived exosomes are a source of shared tumor rejection antigens for CTL cross-priming. Nat Med.

[7] André F, Schartz NEC, Chaput N, Flament C, Raposo G, Amigorena S (2002). Tumor-derived exosomes: a new source of tumor rejection antigens. Vaccine.

[8] Clayton A, Mason MD (2009). Exosomes in tumour immunity. Curr Oncol.

[9] Lee E-Y, Park K-S, Yoon YJ, Lee J, Moon H-G, Jang SC (2012). Therapeutic effects of autologous tumor-derived nanovesicles on melanoma growth and metastasis. PLoS One.

[10] Zhang Y, Luo C (2010). Exosomes derived from IL-12-anchored renal cancer cells increase induction of specific antitumor response in vitro: a novel vaccine for renal cell carcinoma. Int J Oncol.

[11] Bu N, Wu H, Sun B, Zhang G, Zhan S, Zhang R (2011). Exosome-loaded dendritic cells elicit tumor-specific CD8+ cytotoxic T cells in patients with glioma. J Neurooncol.

[12] Andre F, Schartz N, Movassagh M (2002). Malignant effusions and immunogenic tumour-derived exosomes. Lancet.

[13] Hegmans JPJJ, Hemmes A, Aerts JG, Hoogsteden HC, Lambrecht BN (2005). Immunotherapy of murine malignant mesothelioma using tumor lysate-pulsed dendritic cells. Am J Respir Crit Care Med.

[14] Hegmans JP, Veltman JD, Lambers ME, De Vries IJM, Figdor CG, Hendriks RW (2010). Consolidative dendritic cell-based immunotherapy elicits cytotoxicity against malignant mesothelioma. Am J Respir Crit Care Med.

[15] Bard MP, Hegmans JP, Hemmes A, Luider TM, Willemsen R, Severijnen L-A (2004). Proteomic analysis of exosomes isolated from human malignant pleural effusions. Am J Respir Cell Mol Biol.

[16] Hegmans JPJJ, Bard MPL, Hemmes A, Luider TM, Kleijmeer MJ, Prins J-B (2004). Proteomic analysis of exosomes secreted by human mesothelioma cells. Am J Pathol.

[17] Clayton A, Mitchell JP, Court J, Mason MD, Tabi Z (2007). Human tumor-derived exosomes selectively impair lymphocyte responses to interleukin-2. Cancer Res.

[18] Théry C, Amigorena S (2006). Isolation and characterization of exosomes from cell culture supernatants and biological fluids. Curr Protoc Cell Biol.

[19] Lutz MB, Kukutsch N, Ogilvie AL, Rössner S, Koch F, Romani N (1999). An advanced culture method for generating large quantities of highly pure dendritic cells from mouse bone marrow. J Immunol Methods.

[20] Liu JP, Hsieh YZ, Wiesler D, Novotny M (1991). Design of 3-(4-carboxybenzoyl)-2-quinolinecarboxaldehyde as a reagent for ultrasensitive determination of primary amines by capillary electrophoresis using laser fluorescence detection. Anal Chem.

